# Effects of age and radiation treatment on function of extrinsic tongue muscles

**DOI:** 10.1186/s13014-014-0254-y

**Published:** 2014-12-04

**Authors:** John A Russell, Nadine P Connor

**Affiliations:** University of Wisconsin School of Medicine and Public Health, Otolaryngology Head and Neck Surgery, Madison, WI 53706 USA; Department of Communication Sciences and Disorders, University of Wisconsin-Madison, Madison, WI 53706 USA

## Abstract

**Background:**

Radiation treatment for head and neck cancer often results in difficulty swallowing. Muscle weakness and fibrosis have been identified clinically as possible etiologies for swallowing problems following radiation. Aging may compound the effects of radiation on swallowing because radiation-induced damage to muscles and other tissues critical for the oropharyngeal swallow is overlaid on a declining sensorimotor system. However, there have been no investigations of the manner in which aging and radiation treatment effects combine to impact tongue muscles, which are critical effectors of the oropharyngeal swallow.

**Methods:**

Thirty-seven male Fisher 344/Brown Norway rats were divided into four groups; young adults (9 month old), old (32 months old), young radiation (9 months), and old radiation (32 months old). Two fractions of 11 Gy on consecutive days was delivered by external beam radiation to the ventral side of the rat’s body over the anterior portion (20 X 30 mm area) of the anterior digastric muscle. Two-way analysis of variance (ANOVA) was used to examine the effects of age and radiation and their interaction on muscle contractile properties. Post-hoc testing was completed using Fisher’s least significant differences (LSD).

**Results:**

Radiation was associated with a significant decrease in tongue force production and reduced speed of tongue muscle contraction. However, radiation treatment did not lead to muscle atrophy and fibrosis formation in the GG muscle. Radiation treatment did not exacerbate atrophic changes observed with aging, or lead to additional fibrosis formation in the GG muscle from that observed in the other groups.

**Conclusions:**

The purpose of this research was to determine the effect of radiation on muscles of the tongue and to determine whether aging altered the extent of radiation injury to tongue muscles. Radiation was associated with a significant decrease in tongue force production and reduced speed of tongue muscle contraction, and the reduction in the speed of tongue muscle contraction was exacerbated in the aged-rat tongue. This work provides a foundation for future investigations of treatments for concurrent effects of aging and radiation on muscles of the tongue and swallowing.

## Background

Current treatment of head and neck cancer includes a combination of surgery, radiotherapy, and chemotherapy. Unfortunately, even with highly conformal new radiation therapy technologies, a consequence of radiation treatments is the exposure of healthy normal tissues within the radiation field [1,2]. There are many regional complications related to radiation-induced damage to normal tissue of the head and neck, including mucositis, pain, dermatitis, xerostomia, dysphonia, trismus, weight loss, fibrosis, osteoradionecrosis, and dysphagia [3-12]. Dysphagia can be a potentially life-threatening effect of radiation therapy.

Radiation treatment to the head and neck has many negative effects on the swallowing process, including poor pharyngeal motility, epiglottis immobility, reduced laryngeal excursion, poor closure of laryngeal vestibule, poor tongue base function, and aspiration [8,13-17]. Post-radiotherapy swallowing disorders may be primarily attributed to muscular fibrosis, edema, and loss of sensation [18]. Clinical studies suggest that muscle fibrosis, the formation of excess fibrous connective tissue, is the cause of abnormal motility of muscles associated with deglutition post radiation [19]. Fibrosis in the head and neck of humans following radiation has not been confirmed, but is assumed based on physical examination [20,21]. Because tongue strength is also reduced following head and neck radiotherapy [22,23], it is also assumed that rigidity from fibrosis is overlaid on musculature that is compromised and weakened, resulting in profound movement compromise for deglutition [20,21].

Almost half of the new cases of head and neck cancer diagnosed annually in the United States occur in individuals older than 65 years of age [24,25]. The number of patients with head and neck cancer is increasing, and this increase is occurring against a background of a rapidly aging society [26]. Even without the complication of head and neck cancer and radiation therapy, aging is associated with dysphagia in up to 35% of elderly people [27]. Sensorimotor control of the tongue and alterations in tongue muscle function have been linked with age-related dysphagia, including reductions in lingual pressures and increased fatigue [28-32]. Thus, sarcopenia is likely manifested in cranial sensorimotor systems in a manner that affects lingual sensorimotor control and may also affect swallowing actions.

When the effects of aging are combined with other health concerns, disorders, or diseases, it may be expected that even greater deviations in function will be manifested [33]. However, there have been no investigations of the manner in which aging and head and neck cancer radiation treatment combine to impact the muscles of the tongue, which are critical effectors of the oropharyngeal swallow. The purpose of this research is to determine the effect of radiation on muscles of the tongue and to determine whether aging alters the extent of radiation injury to tongue muscles. We hypothesized that radiation would alter the structure and contractility of extrinsic muscles of the tongue. We also hypothesized that radiation would exacerbate degenerative changes seen with age and lead to further increases in muscle atrophy and fibrosis formation in extrinsic tongue muscles of the aged rat.

## Methods

A total of 37 young adult (9 months old) or old (32 months old) Fischer 344/Brown Norway rats were randomized into radiation or no-radiation groups and were used for analysis of muscle structure and function. Rats in the following groups were followed over the course of 12 weeks: 10 young adult [Y], 9 Old [O], 9 young adult radiated [YR], and 9 old radiated [OR]. This research was performed in accordance with principles specified within the *Guide for the Care and Use of Laboratory Animals*, Eighth Edition, National Research Council, 2011. The animal care and use research protocol was approved by the University of Wisconsin School of Medicine and Public Health Animal Care and Use Committee.

**Figure 1:**
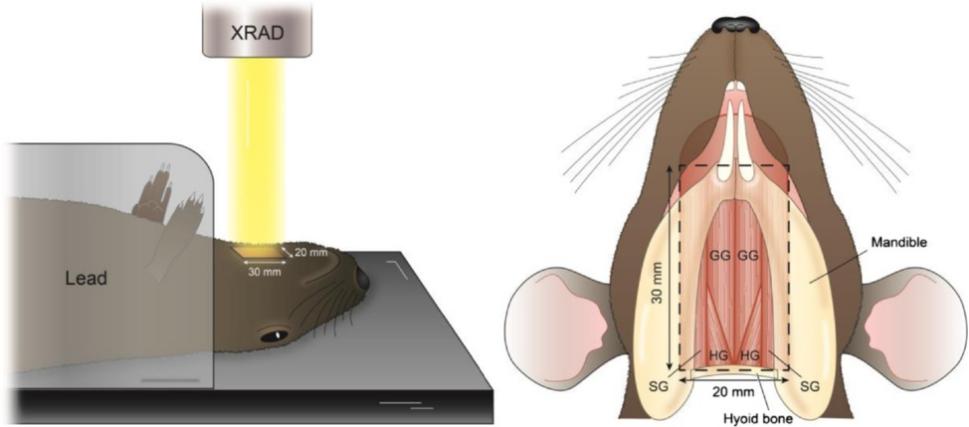
**Illustration of rat position and structures exposed during radiation treatment.**

External beam radiation (XRAD) was used with an acceleration voltage of 320 kVp, a working current of 12.5 mA, and a Thoreaus filter dose rate of approximately 2 Gy/minute. Each rat was placed in 2 mm-thick shielding device that was custom-designed in our laboratory. The shield was used to block radiation exposure to the rat’s body below the hyoid bone. External beam radiation was applied on the ventral side of the rat’s body over the anterior portion of the anterior digastric muscle. The XRAD targeted an approximately 20 mm × 30 mm area (Figure 1).

Previous literature has shown that single doses of 20–30 Gy are associated with changes in muscle morphology and fibrosis, while a single fraction greater than 14 Gy causes irreversible endothelial apoptosis [34-37]. Therefore, this study used the biological effective dose (BED) based on the linear-quadratic formula (BED = E/α = nD (1 + (D/(α/β))) to find an equivalent dose [38,39]. Assuming a α/β ratio for the muscle to be 3, then a single fraction of 16 Gy is equivalent to a biological dose of 103 Gy, which equals a standard fraction of 11 Gy per day for two days. Hence, the radiation action schedule used in this study was 22 Gy over two days and was chosen because of its anticipated biological equivalence to a single fraction of 16 Gy that results in muscle morphology changes, while largely sparing endothelial cells. Therefore, appropriate fraction sizes were used to maintain clinical relevance, limit the potentially confounding endothelium-related radioresponse, and maintain an appropriate radiation schedule for a rat.

Recording of tongue muscle contractile properties was performed over a 1–3 day period following the 12-week post-radiation period. On the day of the experiment, body weights were recorded and rats were anesthetized using intraperitoneal injection of sodium pentobarbital (70 mg/kg). Each rat was placed in a dorsal recumbent position on an operating table under an operating microscope (Zeiss, Thornwood, NY). The hypoglossal nerves were exposed bilaterally using a ventral approach to allow access for the nerve cuff stimulation electrodes. This surgical approach has been documented in previous research literature [40-43]. Core temperature of the rats was monitored at all times and maintained at 37 – 39°C. A small suture was placed into the tip of the tongue for connection to a force transducer (Kent Scientific, Torrington, CT). The stimulation signal and tongue force signal were acquired digitally on a dedicated laboratory computer equipped with a A/D converter (Data Translation, Marlboro, MA) using data acquisition software (Acquire Ver. 1.3.0, Madison, WI).

The tongue was manually protruded from the mouth during the experiment. Optimal direction and line tension on the suture were determined for each rat to yield maximum peak muscle twitch forces. Whole hypoglossal nerves were stimulated bilaterally via the electrode cuffs surrounding the nerve and whole nerve stimulation contractile properties were recorded. These isolated hypoglossal nerve stimulation pulses (1-Hz rectangular-wave pulses, pulse width 0.1 ms) were delivered at supramaximal levels (1.5 times maximum stimulation) to control for small differences in stimulation electrode placement. These stimulation parameters have been reported previously [43,44]. Three 10-sec trials with a 1-min rest period between trials were recorded.

The following measurements were made from retrusive tongue actions generated by whole hypoglossal nerve stimulation: (1) twitch contraction time (CT); the interval (ms) between the onset of stimulation and the point of 50% maximal twitch force, (2) half-decay time (HDT); the interval (ms) between the onset of stimulation and the point of 50% decay from peak twitch force, (3) maximum twitch force; the peak force (mN) generated following a single electrical stimulus, (4) maximum tetanic tension; the fused maximum tetanic force (mN) generated across a range of stimulation frequencies (20 Hz, 40 Hz, 60 Hz, 80 Hz, and 100 Hz), and (5) fatigue ratio; the ratio of average tetanic force (mN) at the end of 2 minutes of stimulation (80 or 100 Hz) relative to the initial tetanic tension (mN), multiplied by 100 to express the value as the percentage of initial tension. As such, a high fatigue ratio indicated a resistance to fatigue [45,46]. In addition, force maxima elicited by stimulation at 1 Hz, 20 Hz, 40 Hz, 60Hz, 80 Hz, and 100 Hz were used to produce a series of curves expressed as a percentage of maximum tetanic force. The curves were fit with a 4-parameter logistic curve using the equation: y = y_0_ + a/1 + (x/x_0_)^b^.

Following whole-hypoglossal nerve stimulation, a 2–3 mm section of the lateral branch of the hypoglossal nerve was removed bilaterally. The rat was repositioned to optimize direction and line tension for forthcoming elicited protrusive tongue actions. Following a 45-min stabilization period, the hypoglossal nerves were stimulated again, but due to the lateral hypoglossal branch section, only the medial branch was effectively stimulated and protrusive muscle contractile properties were measured. The same measurements were made for medial nerve stimulation contractions as described previously.

### Muscle morphology

Following muscle contractile property data collection, the anesthetized rat was euthanized by an overdose of Beuthanasia. The GG muscle on each side was quickly extracted and suspended in oxygenated Ringers solution. The extensor digitorum longus (EDL) of the leg was extracted as a control muscle located outside of the radiation plane. Muscles were wrapped in foil, labeled, frozen in liquid nitrogen, and then stored in a −80°C freezer for further processing.

Serial 10 μm cross-sections were made from the mid-section of the GG muscle using a −20°C cryostat (Leica CM 1850; Meyer Instruments, Houston, TX). Every third section was collected and three sections were mounted per slide and stained for analysis of collagen content and muscle fiber cross-sectional area. Masson’s trichrome staining was used to measure the amount of collagen content (fibrosis) and cross sectional area (fiber size) of the GG muscle. Assays were performed using methods described in previous studies [47]. Briefly, frozen sections were incubated in Bouin’s solution overnight. Slides were rinsed in running tap water to remove picric acid (typically 10–15 min) until the yellow coloration was eliminated. Sections were then stained with iron hematoxylin solution for 10 minutes. Running water was applied for 10 minutes to allow the hematoxylin to turn blue. The sections were then stained with Bierbich’s Scarlet Acid Fuchsin for 10 minutes. After incubation with phosphotingstic/phosphomolybdic acid, the sections were directly transferred into aniline blue solution and maintained for 10 minutes. The sections were dehydrated with 95% and 100% ethanol, cleared with Histoclear, and mounted on slides using Histomount Mounting Solution (National Diagnostics, Atlanta). This procedure stained nuclei black, muscle red, and collagen blue.

### Image analysis

**Figure 2:**
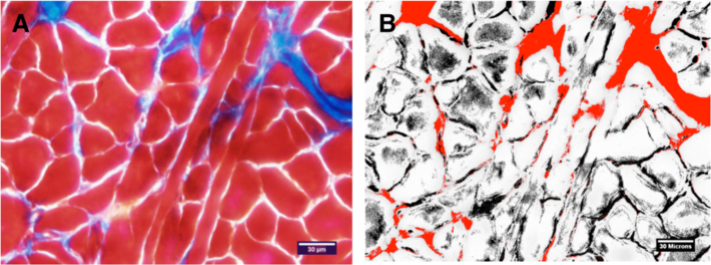
**A representative image of the GG muscle (A) and the same sample separated into a 3-channel hue, saturation, brightness (HSB) stack as described in the methods (B).** The processing algorithm represented the muscle fibers as grey in color, while the collagenous tissue remained blue from the staining process. The threshold was adjusted for the hue channel so that the collagenous tissue area (red) matched that found in the original image (blue).

Tissues were imaged with a fluorescent microscope (BX60, Center Valley, PA). Images were analyzed with ImageJ (Image Processing and Analysis in Java, NIH) and HCImage (Hamamatsu, Bridgewater, NJ). An individual masked to treatment condition (MH) analyzed the images for percent fibrosis area and muscle cross sectional area (CSA). A minimum of .44 mm^2^ was captured and analyzed for percent collagen area from the GG muscle. For analysis of collagen, images were converted into a 3-channel HSB stack (hue, saturation, brightness) that allowed for better separation of the red and green color spectrum. The processing algorithm caused the muscle fibers to appear grey, while the collagenous tissue remained blue from the staining process. The threshold was adjusted for the hue channel so that the collagenous tissue area (blue) matched that found in the original image. A representative image of fibrosis analysis is shown in Figure 2. The collagen area fractions (%) were obtained for analysis of fibrosis. GG muscle CSA (μm^2^) was obtained using a morphological analysis package, HCImage (Hamamatsu, Bridgewater, NJ). Muscle fibers that were only partially included within the image frame were excluded from analysis.

Data are reported as mean and standard error (SE). A two-way analysis of variance (ANOVA) and analysis of covariance with a body weight covariate were used to examine the effects of age and radiation and their interaction on muscle contractile properties (contraction time, half-decay time, maximum twitch tension, tetanic tension, fatigue ratio). In addition, two-way repeated measures ANOVA was used to examine the effects of age, radiation, and their interaction at each stimulation frequency (1 [twitch], 20, 40, 60, 80, & 100 Hz) expressed as a percentage of maximum tetanic force. Post-hoc testing was completed using Fisher’s least significant differences (LSD).

## Results

### Weight loss after radiation treatment

There was a significant interaction between time of weight acquisition (baseline or 12 weeks post radiation treatment) and age (F_[1,33]_ = 35.16, p < .001). Post hoc tests revealed that within the old group, rats receiving radiation treatment lost significantly more weight compared with the control group (p < .001). In addition, within the control condition, the old rats weighed more than the young adult rats at baseline (p < .001).

There was a significant interaction between age and radiation on final weights at 12 weeks (F_[1,33]_ = 6.76, p = .014). In the old group, the radiation group weighed significantly less than the control group (p < .005). In addition, in the control condition, the old rat group weighed significantly more than the young adult group. (p < .001).

### Maximum tetanic force

**Figure 3:**
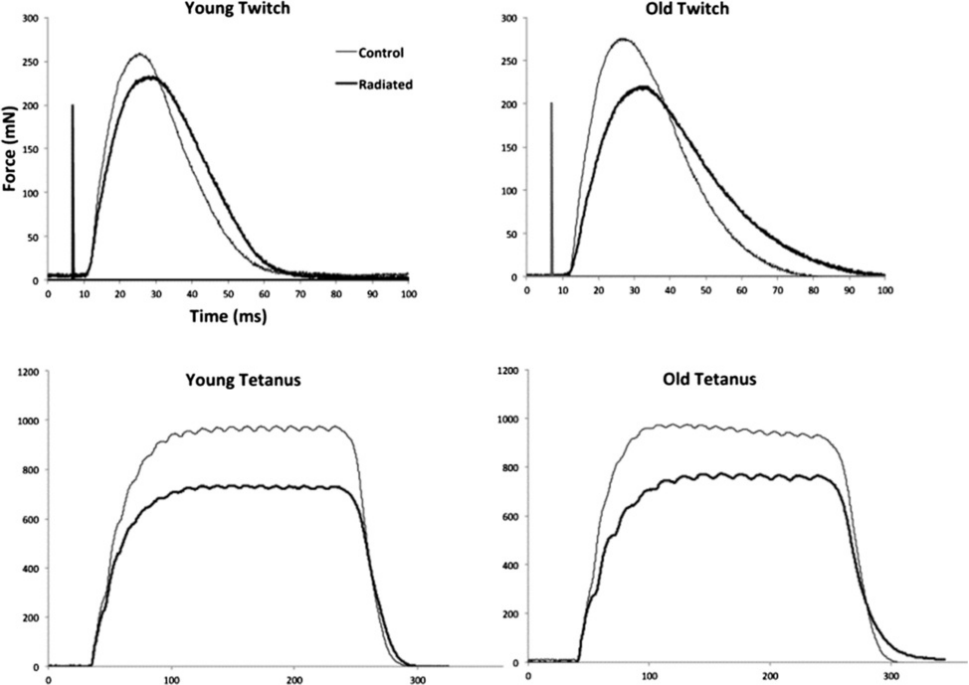
**Representative twitch and tetanic contractions from young adult control, young adult radiated, old control, and old radiated rat.** These examples are representative of group findings, with smaller tetanic and twitch contraction magnitudes (mN), longer CT (ms) and longer HDT (ms) in radiated rats. Age effects are also demonstrated here with longer CTs and HDTs, as well as reduced twitch forces in the old rats.

**Figure 4:**
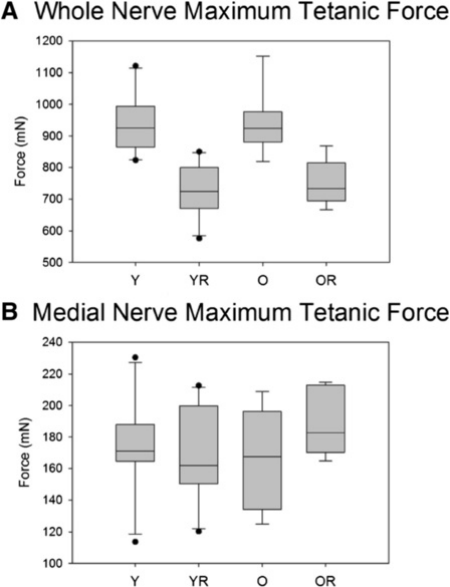
**Maximum tetanic force.** Box and whisker plot showing that **(A)** radiation treatment was associated with significantly (p<.001) reduced maximum tetanic tongue force during whole nerve stimulation in both age groups, and **(B)** that age and radiation did not have a significant effect on maximum tetanic force during medial nerve stimulation. Boxes depict the interquartile range (IQR), with a line at the median. Whiskers extend to the last observation within 1.5x the IQR. The closed circles are observations beyond 1.5x the IQR. Y = Young Adult Control; YR = Young Adult Radiated; O = Old Control, OR = Old Radiated.

Stimulation of the whole hypoglossal nerves bilaterally elicited a retrusive tongue action. Representative retrusive tetanic waveforms for young and old rats in the control and radiation condition are shown in Figure 3. Weight had a significant effect on maximum retrusive tetanic force and, therefore, the results of ANCOVA using a weight covariate are reported. There was not a significant interaction between age and radiation treatment. However, as shown in Figure 4A, there was a significant main effect for age on maximum retrusive tetanic force (F_[1,32]_ = 7.43, p < .01). Based on the weight-adjusted means, the old adult group produced significantly less maximum retrusive tetanic force than the young adult control (p < .001). In addition, there was a significant main effect for radiation treatment with significantly reduced tetanic tension in the radiation treatment group (F_[1,32]_ = 18.62, p < .001).

Selective stimulation of the medial branch of the hypoglossal nerves was performed after sectioning of the lateral branch bilaterally. This stimulation procedure evoked a protrusive tongue action. There was not a significant interaction effect between age and radiation treatment on maximum protrusive tetanic force (F_[1,33]_ = 2.77, p = .11; Figure 4B). In addition, there were not significant main effects for age or radiation treatment (F_[1,33]_ = .59, p = .45 and (F_[1,33]_ = .75, p = .39, respectively).

### Twitch force

**Figure 5:**
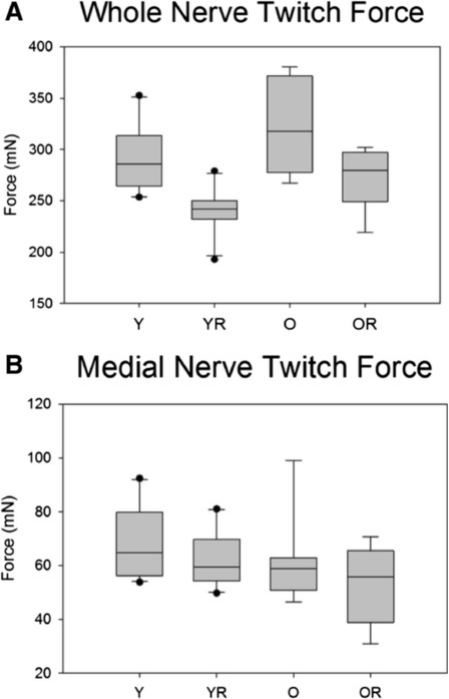
**Twitch force.** Box and whisker plot showing that **(A)** radiation treatment was associated with significantly (p = .01) reduced medial nerve twitch tongue force in both age groups, and **(B)** that age had a significant (p = .04) effect on medial nerve twitch force. Boxes depict the interquartile range (IQR), with a line at the median. Whiskers extend to the last observation within 1.5x the IQR. The closed circles are observations beyond 1.5x the IQR. Y = Young Adult Control; YR = Young Adult Radiated; O = Old Control, OR = Old Radiated.

Weight had a significant effect on retrusive twitch force and, therefore, ANCOVA results using a weight covariate are reported. There was not a significant interaction between age and radiation treatment on twitch force during the retrusive action of the tongue evoked by 1 Hz whole nerve stimulation. In addition, there was not a significant main effect for age (F_[1,32]_ = .50, p = .48). However, there was a significant main effect for radiation treatment (F_[1,32]_ = 6.73, p = .01; Figure 5A). Representative retrusive twitch contraction waveforms for young and old rats in the two conditions are shown in Figure 3

There was not a significant interaction effect between age and radiation treatment on protrusive action of the tongue evoked by 1 Hz medial nerve stimulation. However, there was a significant main effect for age, such that the old group had reduced protrusive tongue twitch forces (F_[1,33]_ = 4.382, p = .04; Figure 5B).

### Contraction time

**Figure 6:**
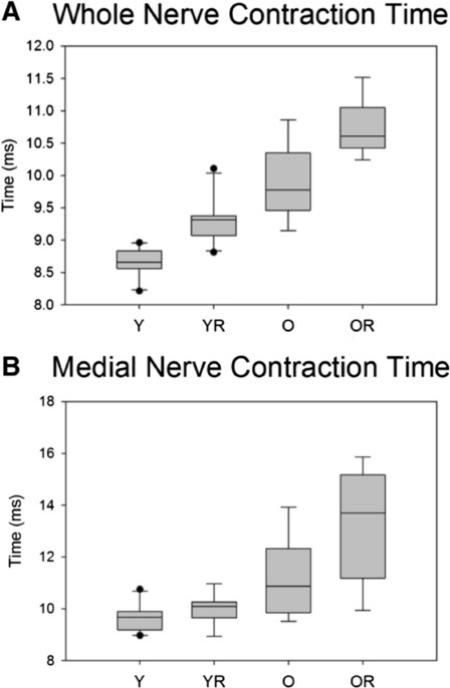
**Contraction time.** Box and whisker plot showing that **(A)** the old group had a significantly (p<.001) longer contraction time and radiation treatment was associated with significantly (p<.001) longer contraction time in both age groups with medial nerve stimulation, and **(B)** that both age and radiation were associated with significantly (p<.001 and p = .009, respectively) longer contraction times with medial nerve stimulation. Boxes depict the interquartile range (IQR), with a line at the median. Whiskers extend to the last observation within 1.5x the IQR. The closed circles are observations beyond 1.5x the IQR.Y = Young Adult Control; YR = Young Adult Radiated; O = Old Control, OR = Old Radiated.

There was not a significant interaction effect observed between age and radiation treatment on contraction time during whole hypoglossal nerve stimulation. However, there was a significant main effect for age with longer contraction times found in the old group (F_[1,33]_ = 103.75, p < .001). In addition, there was a significant main effect for radiation treatment (F_[1,33]_ = 32.859, p < .001). In both young adult and old groups, radiation treatment was associated with significantly increased muscle contraction times (p = .001 and p < .001, respectively). Further, in both control and radiated conditions, the old groups had significantly longer contraction times (p < .001 and p < .001, respectively; Figure 6A).

There was not a significant interaction effect between age and radiation treatment on contraction time during medial nerve stimulation. However, there were significant main effects for age (F_[1,33]_ = 30.44, p < .001) and radiation treatment (F_[1,33]_ = 7.64, p = .009). In both the control and radiation conditions, the old group had significantly longer contraction times than the young adult group (p = .013 and P < .001, respectively). Radiation treatment was associated with a significant increase in protrusive contraction time in the old group (p = .004; Figure 6B).

### Half-decay time

**Figure 7:**
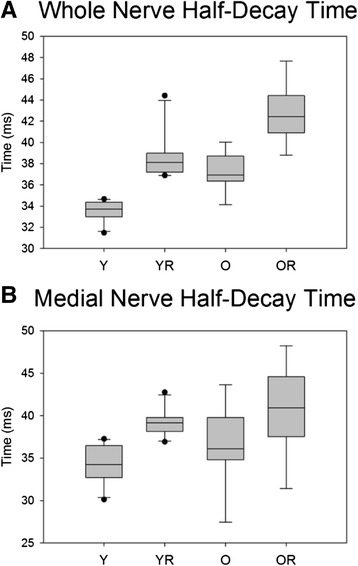
**Half decay time.** Box and whisker plot showing that **(A)** the old control group had significantly (p<.001) longer half-decay time than the young control group with whole nerve stimulation, and **(B)** radiation treatment was associated with significantly (p<.001) longer half decay time in both age groups with medial nerve stimulation. Boxes depict the interquartile range (IQR), with a line at the median. Whiskers extend to the last observation within 1.5x the IQR. The closed circles are observations beyond 1.5x the IQR. Y = Young Adult Control; YR = Young Adult Radiated; O = Old Control, OR = Old Radiated.

There was not a significant interaction effect between age and radiation treatment on half-decay time during whole hypoglossal nerve stimulation. However, there was a significant main effect for age with longer half-decay times found in the old group (F_[1,33]_ = 35.87, p < .001). In addition, there was a significant main effect for radiation treatment on half-decay time (F_[1,33]_ = 66.51, p < .001). In both young adult and old groups, radiation treatment was associated with a significant increase in half-decay time (p = .001 and p < .001, respectively). Further, in both control and radiated conditions, the old groups had significantly longer half-decay times (p < .001 and p < .001, respectively; Figure 7A).

There was not a significant interaction effect between age and radiation treatment on half-decay time during medial nerve stimulation evoked protrusive actions of the tongue (F_[1,33]_ = .10, p = .745). In addition, there was not a significant main effect for age (F_[1,33]_ = 2.519, p = .122). However, there was a significant main effect for radiation treatment on half-decay time (F_[1,33]_ = 14.429, p < .001). Within both the young adult and old groups, radiation treatment was associated with significantly longer half-decay times than the control condition (p = .005 and p = .002, respectively; Figure 7B).

### Force-frequency relationship

**Figure 8:**
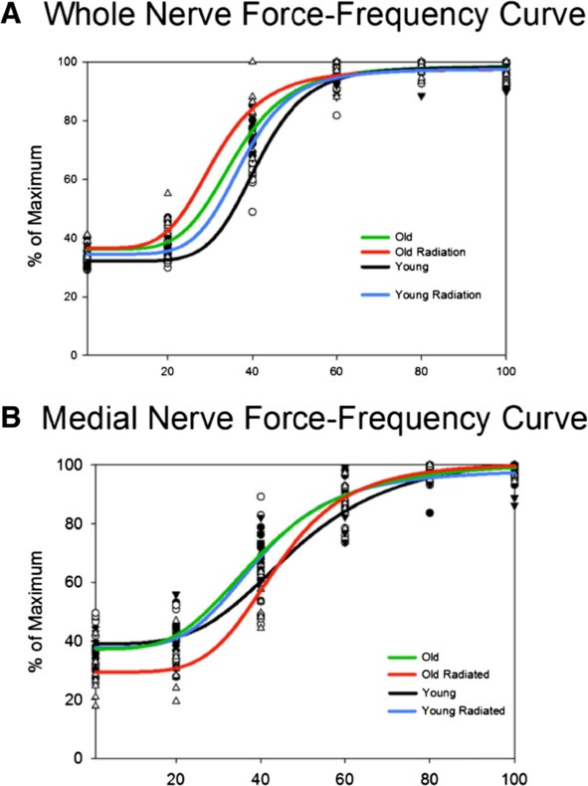
**Force-frequency relationship curves.** Each symbol represents one rat; Old (filled circle), Old radiation (open triangle), Young (open circle), Young radiation (filled triangle). **(A)** Whole nerve stimulation Old r^2^= .98, Old Radiation r^2^ = .96, Young r^2^ =.98, and Young radiation r^2^ = .98 and **(B)** Medial nerve stimulation Old r^2^ = .96, Old Radiation r^2^ = .96, Young r^2^ =.91, and Young radiation r^2^ =.95.

As shown in Figure 8, curves for the old and radiation treatment groups were shifted to the left relative to the young adult controls during bilateral stimulation of the whole hypoglossal nerves. That is, a larger percentage of maximum force was achieved at lower stimulation frequencies and the total force range appeared constrained. There was a significant interaction effect between age and radiation treatment on force-frequency relationship (F_[15, 171]_ = 5.56, p < .001; Figure 8A). Post hoc tests revealed that significant age effects (Young Adult vs. Old; Young Radiation vs. Old Radiation) and significant radiation treatment effects (Young Adult vs. Young Radiation; Old vs. Old Radiation) were found at 20 Hz and 40 Hz.

Stimulation of the medial nerve branches resulted in left-shifted curves for the old control and young radiation groups relative to young adult controls. However, the old radiation group had a curve characterized by a shift to the right, indicating that less force was generated at lower frequencies relative to controls. Therewas a significant interaction effect between age and radiation treatment on force-frequency relationship (F_[15, 171]_ = 4.418, p < .001; Figure 8B). Post hoc tests revealed that significant age effects (Young Adult vs. Old; Young Radiation vs. Old Radiation) were found at 1 Hz, 20 Hz, 40 Hz, and 60 Hz, while significant radiation treatment effects (Young Adult vs. Young Radiation; Old vs. Old Radiation) were found at 1 Hz, 20 Hz and 40 Hz.

### Fatigue

There was a significant interaction effect between age and radiation treatment on fatigue ratio during the whole nerve stimulation of the tongue (F_[1,33]_ = 5.262, p < .028). Post hoc testing revealed that within the young adult group, radiation treatment was associated with lower fatigue ratio (thus, greater fatigue) than the controls (p = .002). In addition, within the control condition, the old group had a significantly lower fatigue ratio (thus, greater fatigue) than the young adult group (p < .001).

There was not a significant interaction effect between age and radiation treatment on fatigue ratio during protrusive tongue actions (F_[1,33]_ = .127, p = .72). However, there was a significant main effect for radiation such that the radiation group manifested significantly reduced fatigue ratios, thus demonstrating increased fatigue for protrusive tongue actions (F_[1,33]_ = 4.704, p = .038).

### Muscle morphology

Muscle fiber cross sectional area (CSA) in the GG muscle was measured in a total of 2067 muscle fibers. On the average, 55 muscle fibers from each rat were measured with a minimum of 40 muscle fibers per rat, allowing calculation of average muscle fiber CSA and standard deviation in muscle fiber CSA per rat. Muscle fiber CSAs were also sorted for each rat by magnitude to allow analysis of the largest 20 muscle fibers per rat. This latter analysis allowed examination of muscle fiber CSA when constrained to a particular phenotype. In addition, muscle fiber CSA in the extensor digitorum longus (EDL) muscle was measured and analyzed to allow a comparison between the groups of a muscle found outside the field of radiation.

**Figure 9:**
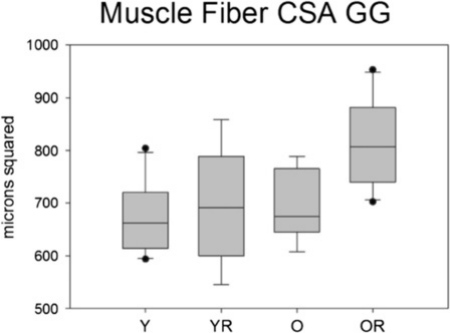
**Muscle fiber cross sectional area (CSA).** Box and whisker plot showing that the old radiation animals had significantly (p=.001) larger muscle fiber CSA. Boxes depict the interquartile range (IQR), with a line at the median. Whiskers extend to the last observation within 1.5x the IQR. The closed circles are observations beyond 1.5x the IQR. Y = Young Adult Control; YR = Young Adult Radiated; O = Old Control, OR = Old Radiated.

**Figure 10:**
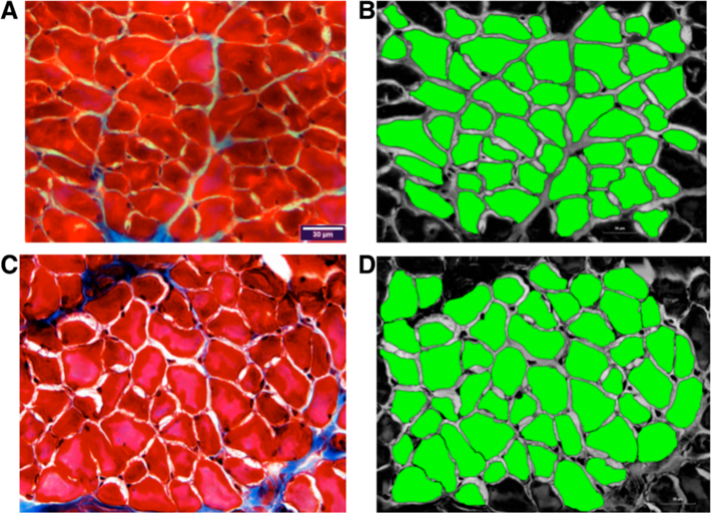
**LEFT: Representative images of genioglossus (GG) muscle CSA from old control (A) and old radiation (C) rats and, RIGHT: corresponding thresholded images used in measurement from these same samples (Old control = B; old radiation = D).** The average muscle CSA for these examples was 657 μm^2^ and 766 μm^2^, respectively.

Weight had a significant effect on average muscle fiber cross-sectional area, and therefore ANCOVA results are reported. There was not a significant interaction effect between age and radiation treatment on the average muscle fiber CSA (F_[1,32]_ = 2.97, p = .094). In addition, there was not significant main effect for age (F_[1,32]_ = 2.34, p = .13). However, there was a significant main effect for radiation treatment (F_[1,32]_ = 7.70, p = .001). The old radiated group had a significantly larger average muscle fiber CSA than the old control group (p = .003; Figure 9) Representative images of genioglossus muscle CSA of old and old radiation groups are shown in Figure 10.

**Figure 11:**
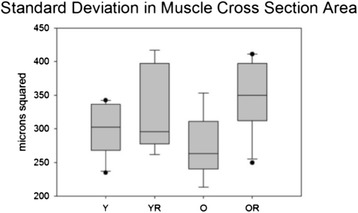
**Standard deviation in muscle fiber cross sectional area (CSA).** Box and whisker plot showing that radiation treatment caused a significant (p<.001) increase in the SD of muscle fiber CSA. Boxes depict the interquartile range (IQR), with a line at the median. Whiskers extend to the last observation within 1.5x the IQR. The closed circles are observations beyond 1.5x the IQR. Y = Young Adult Control; YR = Young Adult Radiated; O = Old Control, OR = Old Radiated.

Weight also had a significant effect on standard deviation in cross-sectional area therefore ANCOVA results are reported. There was not a significant interaction effect between age and radiation treatment on standard deviation of muscle fiber cross sectional area (F_[1,32]_ = 1.759, p = .19). However, there were significant main effects for age (F_[1,32]_ = 4.61, p = .04) and radiation treatment (F_[1,32]_ = 14.31, p < .001) with larger standard deviations found in the old and radiated groups. Specifically, the old radiated group had a significantly larger standard deviation in muscle fiber CSA than the old control group (p = .003; Figure 11).

### Fibrosis

**Figure 12:**
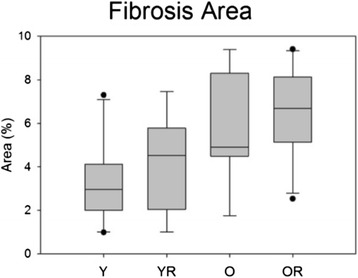
**Fibrosis area.** Box and whisker plot showing that the old groups had increased (p = .003) percent of fibrotic area in the GG muscle. Boxes depict the interquartile range (IQR), with a line at the median. Whiskers extend to the last observation within 1.5x the IQR. The closed circles are observations beyond 1.5x the IQR. Y = Young Adult Control; YR = Young Adult Radiated; O = Old Control, OR = Old Radiated.

There was not a significant interaction effect between age and radiation treatment on the percent fibrosis area (F _[1,33]_ = .977, p = .977). In addition, there was not a significant main effect for radiation treatment (F _[1,33]_ = 1.718, p = .199). However, there was a significant main effect for age with larger areas of fibrosis found in the old group (F_[1,33]_ = 10.71, p = .003; Figure 12).

### Average muscle fiber cross-sectional area of EDL muscle

There was not a significant interaction effect between age and radiation treatment on average muscle fiber CSA in the EDL muscle of the hind limb (F_[1,33]_ = 1.562, p = .22). There was not a significant main effect for radiation treatment in this control muscle (F_[1,33]_ = .077, p = .78). However, there was a significant main effect for age with smaller cross sectional areas in the old group (F_[1,33]_ = 18.21, p < .001).

## Discussion

The purpose of this research was to determine the effects of aging, radiation treatment, and their interaction on the structure and function of the extrinsic tongue muscles. This issue has relevance for human health because radiation treatment is widely used in the care of patients with head and neck cancer with often devastating effects on swallowing function [8,13-17]. Aging may compound the effects of radiation on swallowing because radiation toxicities may be overlaid on a neuromuscular system that also evidences substantial decline in tongue muscle structure and function, with associated functional deficits in swallowing [28-30,48,49]. Because muscle weakness and fibrosis are possible etiologies for post-radiation dysphagia [12,18,19].

The hypothesis of this study was that radiation would alter the structure and contractility of extrinsic muscles of the tongue. In addition, we hypothesized that radiation would exacerbate degenerative changes seen with age and lead to further increases in muscle atrophy and fibrosis formation in extrinsic muscles of the aged rat. The results supported these hypotheses in part: (1) radiation was associated with a significant decrease in tongue force production and reduced speed of tongue muscle contraction; (2) however, contrary to the hypothesis, radiation treatment did not lead to muscle atrophy and fibrosis formation in the GG muscle; (3) also contrary to the hypothesis, radiation treatment did not exacerbate atrophic changes observed with aging, or lead to additional fibrosis formation in the GG muscle from that observed in the other groups.

Aging had significant effects on the muscle contractile properties of retrusive and protrusive actions of the tongue. Protrusive and retrusive tongue actions are both involved in the oral preparatory and oral phases of swallow, with the GG muscle active in humans through the full duration of the oropharyngeal swallow [50]. Altered muscle contractile properties for protrusive actions of the tongue have been reported previously in the aging rat model [51,52]. This study demonstrated that retrusive tetanic contraction force was significantly reduced in the old group. In agreement with previous findings, aging was also associated with significant increases in duration of retrusive and protrusive twitch contraction time and decay time [43]. These alterations in temporal properties of tongue muscle contraction may have functional consequences. It is well known that older individuals swallow more slowly [28] and these increased oropharyngeal transit times are associated with reduced maximum lingual pressures in elderly people [28-30,48,49]. As such, the effect of age on tongue contraction and half decay times reported in the current study suggest that temporal properties of tongue muscle contraction may have an important role in functional tongue movements that equals the importance of tongue force reductions. Thus, the speed and force decrements during the protrusive actions of the tongue seen with aging may have a significant impact on the functional swallow.

Radiation was associated with reductions in both whole nerve maximum tetanic and twitch forces in both young adult and old groups. In addition, radiation had a significant effect on the speed of whole nerve twitch contractions, manifested as increased contraction time and half decay time in both the young adult and old groups. However, speed of contractions was most affected in the old radiated group, showing the slowest contraction times and half decay times of all the groups. Thus, radiation appeared to exacerbate age-related changes in temporal factors of tongue muscle contraction. In human studies, reduction in tongue strength has been associated with radiation therapy [22,23]. In addition, slower oropharyngeal transit times have been shown in human studies following radiation therapy [14]. When reduced force production following radiation is combined with drastic increases in tongue muscle contraction times, as seen in the old rats in this study, functional movements for swallowing could be adversely affected and this may be an underlying cause of dysphagia in older people who receive radiation treatment of the head and neck. However, functional swallow outcomes were not observed or measured in the present study and should be considered for future study.

There are two major factors that determine the shape and position of a force-frequency curve. First, the degree of activation of the muscle at low stimulation frequencies, which is best thought of as the twitch-to-tetanic ratio [53]. Any increase in the twitch force or a decrease in maximum tetanic force will cause the force-frequency relationship to shift to the left. Conversely, if there is a decrease in the twitch force or an increase in maximum tetanic force, then the force-frequency relationship will shift to the right. Second, the contractile speed of the twitch determines at what frequency muscle contraction summation will begin during a stimulation train [53]. A slow-twitch muscle will allow summation of the twitches at lower frequencies, resulting in the force-frequency relationship curve to shift to the left at lower frequencies [54]. Conversely, because fast-twitch muscle will not reach summation at lower frequencies, the force-frequency relationship will shift to the right for these muscle types [55].

During whole nerve stimulation in this study, there was a shift to the left in the force-frequency relationship with age and radiation treatment relative to the curves observed for young control rats. In the case of the retrusive action of the tongue, both the twitch and maximum tetanic forces were reduced in both age groups with radiation treatment. The old control group had a leftward shift in the force-frequency relationship without any reduction in maximum tetanic or increase in twitch force. Therefore, the twitch/tetanic ratio does not appear to be influencing the left shift in the force-frequency relationship. However, with age and radiation there was an increase of the duration of twitch contraction time and half decay time. As shown in Figure 6A, the order of magnitude of average contraction times were: young adult control (shortest contraction time), followed by young adult radiation, old control, and old radiated with the longest contraction times. The order of magnitude of the leftward shift in the force-frequency relationship for each of the groups paralleled the contraction time findings (see Figure 8A). As such, the speed of contraction may have had a substantial influence on the leftward shift in the force-frequency relationship for the stimulated retrusive action of the tongue.

Similarly, during medial nerve stimulation, there was a shift to the left in the force-frequency relationship curves in the young adult radiation group and the old control group from the curves observed for young control group. This leftward shift in the force-frequency relationship can be attributed to the increased contraction times shown for these groups in Figure 8B. However, in the old radiation group, there was a right shift in force-frequency relationship. A rightward shift in the force-frequency relationship might be attributed to a shift in muscle fiber type toward fast-twitch fibers because muscles would not reach summation at lower frequencies and therefore would have lower force production. Although muscle fiber type assays were not performed in this study, previous research has shown that radiation exposure leads to a transformation or replacement to a slower muscle fiber type distribution [56]. Further, the longer contraction times (Figure 6B) in the old radiation group do not support the concept of a shift to a faster fiber type distribution in the GG muscle. There are a few possible explanations for the rightward shift in medial nerve force-frequency relationship beyond fiber type and contraction speed. Radiation treatment can damage the sarcoplasmic reticulum (SR) that could then result in less Ca^2+^ release in response to action potential [57]. Consequently, low frequencies of stimulation may produce lower than normal intracellular Ca^2+^ and thus less force production. However, at higher frequencies, enough Ca^2+^ could be released to reach the same relative force [57]. Another possible explanation could be that radiation decreases membrane excitability such that fewer muscle fibers are activated with a given stimulation frequency [58]. The fact that this rightward shift in force-frequency relationship only occurred in the protrusive action of the old radiation group is not easily explained. However, the method by which the radiation treatment was administered could have possibly exposed the protrusive (GG) extrinsic muscles of the tongue to more radiation than retrusive muscles (SG, HG), because the retrusive muscles were partially shielded from the radiation beam by the mandible (see Figure 1).

In the current study, an aging effect was not seen in GG muscle CSA. This is in contrast to previous findings in the hind limb and human tongue that have shown that muscle atrophy is commonly associated with aging [59-65]. However, the current study is in agreement with previous findings that have shown no changes in GG muscle fiber CSA as a function of age using the current rat model [66]. Therefore, although there was evidence of decreased force production of the tongue in the current study, it is possible that muscle atrophy within the GG muscle was not contributing to force decrements within the 344/Brown Fischer Norway rat. Atrophy in other muscles within the tongue, such as the intrinsic muscles, changes in muscle activation patterns, signal transmission at the neuromuscular junction, or muscle fiber type conversion or loss may be factors contributing to the reduced forces that were not measured in this study.

Previous investigations using fractionated radiation (40 equal fractions over 8 weeks) have shown significantly smaller muscle fibers in the rat hind limb as early as two weeks post-radiation, with fibrosis formation at 6 months post-radiation [67]. A single fraction of 30 Gy to the rat hind limb created cellular alterations and fibrosis within muscle as early as 6 weeks following radiation exposure [37]. In the current study, an increase in muscle fiber cross sectional area with radiation treatments was found. This is not in agreement with previous findings of radiation exposure. It may be possible that the radiation dosage used in this study (2 fractions of 11 Gy) may not have been high enough to cause reductions in muscle fiber CSA. However, it is also possible that there was radiation-induced edema within the muscle fibers. Typically, 2–6 weeks following radiation therapy, acute problems such as inflammation resolve [37]. However, it has been shown that inflammation can occur in the sub-acute phase and radiation myositis has been reported 5 months after radiation treatment [68]. Gallet et al. [37] showed that radiation-induced inflammation maximized at 30 days and then stabilized at 3 months. Therefore, the 12-week post-radiation observation period in the current study may be right on the cusp of the post-inflammatory phase and may possibly explain the increase in GG muscle fiber CSA associated with radiation treatment.

In the current study, aging was associated with an increase in the fibrosis area of the GG muscle. This is in agreement with previous literature that shows an increased amount of fibrotic material in aged muscle [69,70]. However, radiation treatment did not have a significant effect on fibrosis formation in GG muscle. These findings would seem to dispel the linkage of fibrosis formation and dysphagia in humans post-radiation [71]. However, the 12-week post-radiation follow-up period used in this study was shorter than the 6 months to one year that reportedly marks the onset of suspected fibrosis-induced dysphagia reported in human patients [72]. Thus, further follow-up time might be necessary with the rat model to allow comparison of findings in the rat to human conditions.

Constraints of the experimental rat model, such as the need for repeated anesthesia and limited lifespan constrain the number of feasible dose-fractionation schemes that can be employed. However, biologically equivalent dose-fractionation (BED) schemes can be estimated according to the linear-quadratic formula (BED = E/α = nD (1 + (D/(α/β))) to find an equivalent dose [38,39]. Most clinical radiotherapy regimens use fractionation to minimize late normal tissue damage. Conventional radiotherapy for head and neck cancer typically consists of daily fractionation (5 days/week; approximately 2 Gy/fraction) over the course of several weeks (usually 7 weeks) for an overall total dose of 70 Gy and a BED of 116 Gy for normal non-cancerous tissue (α/β ratio 3) [73,74]. In the current study, 2 fractions of 11 Gy per day for an overall dose 22 Gy and a BED of 103 Gy for normal non-cancerous tissue (α/β ratio 3). Previous literature has shown that a single fraction greater than 14 Gy causes irreversible endothelial apoptosis [34-37]. Therefore, appropriate fraction sizes were used to maintain clinical relevance, limit the potentially confounding endothelium-related radioresponse, and maintain an appropriate radiation action schedule for a rat.

Single radiation doses of 20–30 Gy have been associated with development of fibrosis in animal limbs and thus conflict with the results of this study [34,35,37]. A possible explanation is that the radiation dosage of two fractions of 11 Gy (BED, 103 Gy) used in this study was less damaging than the single fractions of 20–30 Gy (BED, 153–300 Gy) given to limbs, a situation necessitated in this study because radiation was delivered to the airway and maintenance of animal health and survival were substantially affected at higher radiation levels in preliminary experiments (data not reported). Accordingly, fibrosis development may not be consistent with the lower dose used here. Further, based on the increased GG muscle CSA found in the radiation treatment groups and the possibility of radiation myositis, it may be possible that the radiation-treated rats were still in the inflammatory phase of healing post-radiation and had not yet progressed to the fibrosis formation stage of wound healing. Thus, future research with longer post-radiation follow up periods may discover fibrosis within the GG or other muscles of the head and neck. Increased radiation exposure to the tongue base, however, may not be a viable option due to issues surrounding survival of the experimental animals.

Interventions for radiation-induced injury to muscles involved in swallowing can be both behavioral and pharmacological. Understanding the underlying causes for swallowing abnormalities observed in individuals who have received radiation therapy allows us to provide a better course of treatment. This work provides a foundation for future investigations of possible treatments and the timing of treatments for the effects of aging and radiation on muscles of the tongue. The ultimate goal of this line of research is improvement in swallowing function and quality of life for older individuals who must undergo head and neck radiation as a part of their clinical care.
